# Association between endothelial nitric oxide synthase (eNOS) gene variants and nitric oxide production in preeclampsia: a case–control study in Ghana

**DOI:** 10.1038/s41598-023-41920-w

**Published:** 2023-09-07

**Authors:** Linda Ahenkorah Fondjo, Enoch Ofori Awuah, Samuel Asamoah Sakyi, Ebenezer Senu, Eric Detoh

**Affiliations:** 1 Department of Molecular Medicine, School of Medicine and Dentistry, Kwame Nkrumah University of Science and Technology, Kumasi, Ghana; 2 Nkawie-Toase Government Hospital, Toase, Ghana

**Keywords:** Genetics, Molecular biology, Diseases, Medical research, Molecular medicine, Pathogenesis

## Abstract

Evidence suggests that a major cause of PE is endothelial dysfunction emanating from the reduced bioavailability of Nitric oxide (NO). Variants of endothelial nitric oxide synthase (eNOS) gene may lead to decreased NO levels. We explored the association between eNOS gene variants and nitric oxide levels among preeclamptic women in the Ghanaian population. This case–control study included 75 preeclamptic women and 75 healthy normotensive pregnant women attending antenatal care at the Nkawie-Toase Government Hospital, Ghana. A well-structured questionnaire was used to collect socio-demographic, obstetric and clinical data. Blood was obtained for DNA extraction; the gene variants were determined using PCR and RFLP. Preeclamptic women had significantly lower NO concentration compared to the normotensives (*p* < 0.0001) and was significantly different between VNTR variants (*p* < 0.0001). A significant difference in VNTR intron 4 distribution was also observed between the preeclamptic and normotensive women with 4c4c” (12.0%) and “4a4c” (1.3%) genotypes found predominantly in preeclamptic women (*p* < 0.0001). There was significantly higher distribution of “TC” genotype in preeclamptic women (44.0%) compared to normotensives (22.7%) (*p* = 0.019). However, possessing “4a4b” (cOR: 0.17, 95% CI 0.04–0.64) and “4b4b” (cOR: 0.09, 95% CI 0.02–0.38) significantly decreased the likelihood of experiencing preeclampsia by 83% and 91% respectively. Nitric oxide is reduced in preeclamptic women. NO levels in preeclampsia are altered by VNTR intron 4 variants but not T786C variants. Possessing VNTR intron 4 “4b” allele decreases the risk of PE while the “4c” allele increases the risk of PE. There is the need for eNOS variant screening and nitric oxide estimation among pregnant women for early prediction of women at risk of preeclampsia.

## Introduction

Preeclampsia (PE) is a major contributor to maternal and neonatal mortality and morbidity worldwide. It complicates about 2–8% pregnancies globally^1^. In developing countries, about 15% of all maternal deaths are attributed to preeclampsia^1^. The prevalence of preeclampsia in Ghana ranges between 5.6 and 6.01%^2,3^. It is has been reported that Ghanaian women are at increased risk of preeclampsia as compared to several other countries^4^.

The exact etiology of preeclampsia is not fully understood; however, several genetic and environmental factors including mutations, family history of hypertension or preeclampsia and obesity have been implicated in the development of preeclampsia^5,6^. The pathophysiology of this multifaceted condition hinges on the production of a number of anti-angiogenic factors which directly affect NO function, causing angiogenic imbalance and impaired vasodilation leading ultimately to endothelial dysfunction. Currently, the pharmacological approaches for treatment and prevention of preeclampsia include the administration of nifedipine, labetalol and low dose aspirin^7^; including magnesium sulfate which is frequently used to prevent seizures^8,9^.

We have earlier reported that altered bioavailability of nitric oxide is a key factor for endothelial dysfunction in preeclampsia^10^. Previous studies have examined the role of genetic variations in the endothelial nitric oxide synthase (eNOS) gene in the development of preeclampsia^11–13^. Some of these linkage studies suggest that polymorphisms in the eNOS gene may modulate NO formation in PE^14,15^. Several variants of the eNOS gene exist, nevertheless, none of these gene variants have been studied in our geographical location. T-786C (rs2070744) and 27 bp VNTR in intron 4, have been reported to be associated with NO formation and concomitantly have significant association with hypertensive disorders in pregnancy^15^. It is thought that these genetic variations in the eNOS gene alter bioavailability of nitric oxide and predisposes women to hypertensive disorders of pregnancy. The eNOS gene is made up of 26 exons on chromosome 7. T-786C (rs2070744), is a single nucleotide polymorphism in the promoter region. An insertion-deletion polymorphism within intron 4 comprises two common alleles, a and b (4 and 5 repeats respectively). It also possesses two less common variants ‘c’ and ‘y’ (6 and 3 repeats respectively). These variants have high minor allele frequencies^11^.

Molecular studies on genetic predisposition of preeclampsia in Ghana are very limited. Moreover, eNOS variants and preeclampsia have not been explored. Likewise, there is limited data on eNOS variants and plasma NO levels in preeclamptic women in sub-Saharan Africa. These warrant for studies in these population. We assessed the association between eNOS gene T-786C (rs2070744) and 27 bp VNTR eNOS variants and nitric oxide levels among Ghanaian preeclamptic women.

## Methods

### Study design and study site

This case–control study was conducted at the Nkawie-Toase Government Hospital. The hospital is a municipal hospital located at Nkawie which is the capital of the Atwima Nwabiagya Municipality in the Ashanti Region of Ghana. The hospital has a maternity unit with a 30-bed capacity and has a vibrant antenatal care (ANC) department that attends to an average of 120 pregnant women on ANC days. The hospital also receives referrals from other health facilities in the municipality; most referrals received by the hospital on maternal health center largely on hypertensive disorders of pregnancy.

### Study population

The study included pregnant women with a confirmed diagnosis of preeclampsia and healthy normotensive pregnant women aged between 18 and 40 years. The preeclamptic women were newly diagnosed preeclamptic cases and were recruited by an Obstetrician/Gynaecologist using the International Society for the Study of Hypertension in Pregnancy (ISSHP) diagnostic criteria^16^. Briefly, the ISSHP guidelines for the diagnosis of preeclampsia stipulate that the condition be diagnosed as gestational hypertension coupled with one or more of new—onset conditions such as proteinuria, any organ dysfunction of the mother and uteroplacental dysfunction. The blood pressure measurement per the criteria for each diagnosed case was ≥ 140 mmHg for systolic pressure and ≥ 90 mmHg for diastolic pressure. Pregnant women who did not give consent to partake in the study were excluded. Pregnant women with history of diabetes mellitus, chronic hypertension, kidney disease, gestational diabetes and cardiovascular disorders such as coronary heart disease, stroke, peripheral arterial disease and aortic disease were also excluded.

### Sample size

The sample size was obtained using the online sample size calculator for matched case–control study (https://www.openepi.com/SampleSize/SSCC.htm). One hundred and fifty (150) pregnant women were recruited for the study, comprising 75 preeclamptic women and 75 healthy normotensive pregnant women.

### Ethical consideration

Ethical approval for the study was obtained from the Committee on Human Research, Publication and Ethics, Kwame Nkrumah University of Science and Technology (CHRPE/KNUST/AP/028/22). Approval was also obtained from the Hospital administration of Nkawie-Toase Government Hospital. Participants were duly informed about the study and consent was obtained after explaining the purpose of the research, benefits, potential harms and confidentiality to the respondents. The study was conducted in accordance with the declaration of Helsinki. Each participant gave their written informed consent after the aim of the study had been explained.

### Questionnaire administration

Data on socio-demographic, obstetric and clinical information were obtained from each participant using a well-structured and validated questionnaire adapted from Tashie et al*.*^10^, with modifications.

### Blood sample collection

About five (5) ml of venous blood was drawn from each participant and used for both biochemical and molecular analysis. Briefly, each participant was made to sit or lie comfortably and sampling area was carefully cleaned with 70% alcohol swab. A tourniquet (Narang Medical Ltd, India) was used to make veins visible. Blood was taken from either the basilic, cephalic or median cubital veins of participants with 21-gauge needles with 5 ml syringes attached (Narang, Medical Ltd, India) and dispensed into serum separator and EDTA tubes (Becton Dickinson, United Kingdom); 2 ml and 3 ml respectively. The blood sample in serum separator tube was centrifuged and serum separated. For each participant, aliquots of approximately 600 µl and 200 µl serum samples were made into two separate 1.5 ml microcentrifuge tubes (New England Biolabs, United Kingdom) for renal function and ELISA respectively. The aliquots were stored at − 20 °C until analysis.

### Biochemical analysis

Serum samples were analysed for nitric oxide (NO) concentration and renal function. For each participant, NO, urea, creatinine, uric acid, magnesium, sodium, potassium and chloride concentrations were assessed.

### Nitric oxide estimation

About 200 µl serum was used to estimate NO using enzyme-linked immunosorbent assay (ELISA) kits (Melson Shangai Chemical Ltd, China), following manufacturer’s protocol. The optical density was read at 450 nm using a microplate reader (RT-2100C microplate reader, Rayto). The levels of nitric oxide in the test samples were calculated from the standard curve plotted using the different standards with known concentrations.

### Renal function test

Renal function was determined using a fully automated analyser (ChemWell-T Analyser, USA). Serum samples was used to estimate concentrations of urea, creatinine, uric acid and electrolytes (sodium, potassium, magnesium and chloride) of study participants. Urea, creatinine, magnesium and uric acid were analysed using reagents from (Elitech Group, France) while the sodium, potassium and chloride were estimated using an Ion Selective Electrode (ISE) analyser (Model XI-921F, Caretium Medical, China, EC Rep; Prolinx GmbH, Germany).

### DNA analysis for T-786C (rs2070744) and 27bp VNTR in intron 4

Genomic DNA was extracted using a modified, simplified non-enzymatic salting-out method. Good quality DNA was obtained with the concentration ranging between 60 and > 100 ng/µl. Extracted DNA was used for analyses of rs2070744 and VNTR in intron 4.

### T-786C polymorphism

Genotypes for the T-786C were determined using the polymerase chain reaction (PCR) and restriction fragment length polymorphism techniques (RFLP). Primer sequences (New England Biolabs, UK) included; 5-TGG AGA GTG CTG GTG TAC CCC A-3 (sense) and 5-GCC TCC ACC CCC ACC CTG TC-3 (antisense).

The PCR reaction was performed in a 25 µl reaction volume in a thermal cycler (Applied Biosystems 2720, UK) and included genomic DNA, 10 µM of each primer, 10 mM of dNTPs, 5X PCR standard reaction buffer, nuclease-free water and DNA Taq Polymerase (New England Biolabs, UK). The volumes of each of the components in the 25 µl PCR reaction volume are as follows; 5 µl of 5X PCR standard reaction buffer diluted to a final concentration of 1X, 0.5 µl of 10 mM dNTPs to a final concentration of 200 µM, 0.5 µl of 10 µM of both forward and reverse primers all to a final concentration of 0.2 µM, 0.125 µl of DNA Taq polymerase to a final concentration of 1.25 units/50 µl, 1 µl of genomic DNA and 17.375 µl of nuclease-free water.

The PCR reaction mixtures were heated to 94 °C for 30 s for initial denaturation and 30 cycles at 94 °C for 15–30 s for denaturation, 45–68 °C for 15–60 s for annealing, and 68 °C for 1 min for extension. Final extension was conducted at 68 °C for 5 min. The amplified PCR products were digested with MspI (New England Biolabs, UK), producing fragments of 140 and 40 bp for the wild-type allele, or 90, 50 and 40 bp in the case of a polymorphic variant. Gel electrophoresis of was used to examine results for the polymorphism with 2% agarose gel.

### Restriction enzyme (MspI) protocol

Amplicons from T-786C PCR reactions were utilized for the enzyme digestion. The reaction mixture contained 15 µl DNA, 5 µl (1X) of 10X rCutSmart buffer, 1.0 µl of MspI restriction enzyme and 29 µl of nuclease free water (New England Biolabs, UK). The enzyme was kept on ice while being used and the final mixture was incubated for about 5–15 min at 37 °C with a heating block (ThermoQ, China).

### VNTR (27 bp-repeat) polymorphism in intron 4

Genotypes for the VNTR polymorphism in intron 4, were determined by PCR and fragment separation by electrophoresis in agarose gel using the primers (New England Biolabs, UK) 5-AGG CCC TAT GGT AGT GCC TTT-3 (sense) and 5-TCT CTT AGT GCT GTG GTC AC-3 (antisense).

The PCR reaction was also performed in a 25ul reaction volume that included genomic DNA, 10 µM of each primer, 10 mM of dNTPs, 5X PCR standard reaction buffer, nuclease-free water and DNA Taq Polymerase (New England Biolabs, UK). The volumes of each of the components in the 25 µl PCR reaction volume are as follows; 5 µl of 5X PCR standard reaction buffer diluted to a final concentration of 1X, 0.5 µl of 10 mM dNTPs (final concentration of 200 µM), 0.5 µl of 10 µM of both forward and reverse primers (final concentration of 0.2 µM), 0.125 µl of DNA Taq polymerase (final concentration of 1.25 units/50 µl), 1 µl of genomic DNA and 17.375 µl of nuclease-free water.

The PCR reaction mixtures were heated to 94 °C for 30 s for initial denaturation and 30 cycles at 94 °C for 15–30 s for denaturation, 45–68 °C for 15–60 s for annealing, and 68 °C for 1 min for extension. Final extension was conducted at 68 °C for 5 min. Gel electrophoresis using 2% agarose gel was employed to visualize the alleles. Fragments of 339, 393, 420 and 447 bp correspond to the eNOS alleles 4y, 4a, 4b, and 4c, respectively.

### Agarose gel electrophoresis

**Figure 1:**
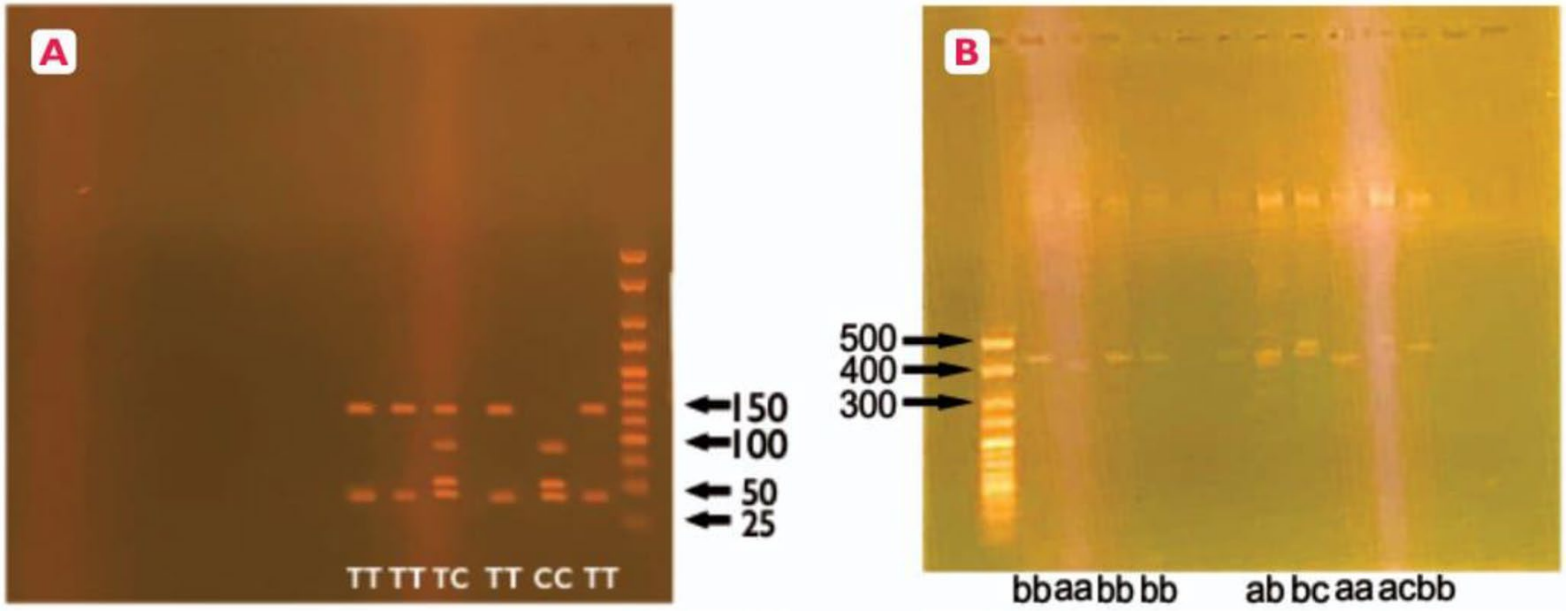
Agarose gel electrophoresis of T786C variants (**A**) and VNTR variants (**B**). (**A**) PCR–RFLP analysis for detection of T-786C polymorphism using MspI restriction enzyme. Enzyme restriction on − 786 T produces 140 bp and 40 bp fragments. Enzyme restriction on − 786C produces fragments 90 bp, 50 bp and 40 bp. Fragments 140 bp and 40 bp correspond to TT genotype, 90 bp, 50 bp and 40 bp correspond to genotype CC and fragments 140 bp, 90 bp, 50 bp and 40 bp correspond to TC genotype.

The process was conducted by transferring the prepared agarose gel onto the gel tank of the electrophoresis unit (Cleaver Scientific, UK). The tank was filled with TBE buffer with ethidium bromide ensuring it covered the gel completely. 10 µl of the ladder (Meridian Bioscience, USA) was loaded and 10 µl of DNA samples were also loaded. For each of the samples, 2 µl of 6X loading dye (ThermoFisher Scientific, UK) was added. The gel was run at 110 V for 45 min. The gel was then visualized under UV transilluminator (Cleaver Scientific, UK) (Fig. 1).

### Data management and statistical analysis

Collected data was entered, cleaned and coded using Microsoft Excel 2019. All statistical analyses were done using the Statistical Package for Social Sciences (SPSS) Version 26.0 (Chicago IL, USA) and GraphPad prism version 8.0 (GraphPad software, San Diego California USA, www.graphpad.com). Categorical variables were presented as frequency and percentages whilst continuous variables were presented as means and standard deviations or median interquartile range depending on the normality test by Kolmogorov–Smirnov test. Chi-square test was used to determine the difference in sociodemographic characteristics between normal and preeclampsia patients. Difference between normal and preeclampsia patients for parametric continuous variables were determined by independent sample t-test whilst non-parametric continuous variables were determined by Mann–Whitney U-test. Moreover, Chi-square test was used to determine the difference in allele frequency and genotype distribution in normal and preeclamptic pregnant women. The association between allele and nitric oxide concentration among preeclamptic pregnant women was determined by Kruskal–Wallis Test and post-hoc test by Bonferroni test. Logistic regression analyses were used to determine the association between allele and risk of preeclampsia. *P *value of < 0.05 was considered statistically significant.

## Results

### Sociodemographic characteristics of study participants

Most of the preeclamptic women were overweight (56.0%), with 26.7% being in obesity class I group. More than half of the normotensives were also overweight (50.7%) with an equal percentage (18.7%) being normal weight and in the obesity class I group (18.7%). There was a significant association between body mass index and preeclamptic status of participants (*p* = 0.016).

Majority of preeclamptic women were within 26–30 years (28.0%) with 21.3% within 31–35 years and 36–40 years (20.0%). Most of the normotensives however, were within 21–25 years (32.0%), closely followed by 31–35 years (25.3%) and 26–30 years (20.0%), although age group was not significantly associated with preeclamptic status (*p* = 0.0690). Likewise, marital status (*p* = 0.8820), educational level (*p* = 0.5950), ethnicity (*p* = 0.3510), employment status (*p* = 0.2510), occupation (*p* = 0.4560) was not significantly associated with preeclampsia (Table 1).

**Table 1:** Sociodemographic characteristics of study participants.

Variable	Normotensive (n = 75)	Preeclamptic (n = 75)	*p* value
Age group (years)			0.0690
18–20	6 (8.0)	12 (16.0)	
21–25	24 (32.0)	11 (14.7)	
26–30	15 (20.0)	21 (28.0)	
31–35	19 (25.3)	16 (21.3)	
36–40	11 (14.7)	15 (20.0)	
Marital status			0.8820
Single	38 (50.7)	35 (46.7)	
Married	34 (45.3)	37 (49.3)	
Divorced	3 (4.0)	3 (4.0)	
Educational level			0.5950
Primary	13 (17.3)	15 (20.0)	
JHS	6 (8.0)	10 (13.3)	
SHS	29 (38.7)	29 (38.7)	
Tertiary	27 (36.0)	21 (28.0)	
Ethnicity			0.3510
Akan	52 (69.3)	59 (78.7)	
Ewe/Ga	11 (14.7)	6 (8.0)	
Northerner	12 (16.0)	10 (13.3)	
Employment status			0.2510
Unemployed	21 (28.0)	15 (20.0)	
Employed	54 (72.0)	60 (80.0)	
Occupation			0.4560
No formal occupation	21 (28.0)	15 (20.0)	
Informal	34 (45.3)	35 (46.7)	
Formal	20 (26.7)	25 (33.3)	
BMI			**0.0160**
Normal	14 (18.7)	13 (17.3)	
Overweight	38 (50.7)	42 (56.0)	
Obese class I	14 (18.7)	20 (26.7)	
Obese class II	9 (12.0)	0 (0.0)	

*BMI* body mass index, *JHS* junior high school, *SHS* senior high school.

*p* < 0.05 and bolded means statistically significant.

### Obstetric and clinical characteristics of study participants

The preeclamptic women were mostly in the gestational age of 26–30 weeks and 36–40 weeks (30.7%), whereas the normotensives were in the gestational age of 36–40 weeks (36.0%) and 26–30 weeks (32.0%). Also, most preeclamptic women had been pregnant 1–2 times (38.7%) but had not given birth or given birth once (46.7%). Similarly, most normotensives were found to had been pregnant 1–2 times (36.0%) but had not given birth or given birth once (36.0%). However, the there was no significant association between participants gestational age (*p* = 0.7970), gravidity (*p* = 0.4640), parity (*p* = 0.5000) and preeclampsia among study participants. In addition, no significant association was found between experiencing miscarriages (*p* = 0.9350), still births (*p* > 0.9999), preterm birth (*p* = 0.3560), multiple births (*p* = 0.3370) and preeclamptic status among study participants. Furthermore, history of alcoholism (*p* = 0.5600), contraceptive use (*p* = 0.6660), family history of preeclampsia (*p* = 0.0800) and family history of hypertension were not significantly associated with preeclampsia among study participants (Table 2)**.**

**Table 2:** Obstetric and clinical characteristics of study participants.

Variable	Normotensives (n = 75)	Preeclamptic (n = 75)	*p* value
Gestational age (weeks)			0.7970
21–25	8 (10.7)	8 (10.7)	
26–30	24 (32.0)	23 (30.7)	
31–35	16 (21.3)	21 (28.0)	
36–40	27 (36.0)	23 (30.7)	
Gravidity			0.4640
1–2	27 (36.0)	29 (38.7)	
3–4	26 (34.7)	28 (37.3)	
5–6	11 (14.7)	13 (17.3)	
> 6	11 (14.7)	5 (6.7)	
Parity			0.5000
0–1	27 (36.0)	35 (46.7)	
2–3	31 (41.3)	24 (32.0)	
4–5	11 (14.7)	12 (16.0)	
≤ 6	6 (8.0)	4 (5.3)	
Number of miscarriages			0.9350
0	58 (77.3)	58 (77.3)	
1	12 (16.0)	11 (14.7)	
2–3	5 (6.7)	6 (8.0)	
Number of still births			> 0.9999
0	69 (92.0)	69 (92.0)	
1	6 (8.0)	6 (8.0)	
Number of preterm birth			0.3560
0	62 (82.7)	66 (88.0)	
1	13 (17.3)	9 (12.0)	
Number of multiple births			0.3370
0	67 (89.3)	63 (84.0)	
1	8 (10.7)	12 (16.0)	
History of alcoholism	2 (2.7)	1 (1.3)	0.5600
Ever used contraceptive	14 (18.7)	12 (16.0)	0.6660
Type of contraceptive			
Oral pill	11 (78.6)	9 (75.0)	
IUD	3 (21.4)	3 (25.0)	
Family history of PE	0 (0.0)	3 (4.0)	0.0800
Family history of hypertension	11 (14.7)	20 (26.7)	0.0700

*IUD* intrauterine device, *PE* preeclampsia.

### Biochemical characteristics of study participants

The preeclamptic women had significantly lower levels of magnesium (0.75 mmol/L) and potassium (3.76 mmol/L) compared to the normotensives with magnesium and potassium levels of 0.82 mmol/L and 4.13 mmol/L respectively (*p* < 0.0001). Similarly, preeclamptic patients had significantly lower levels of sodium (137.22 mmol/L) and chloride (101.56 mmol/L) compared to normotensives (Na: 140.76 mmol/L, Cl: 102.84 mmol/L).

On the contrary, preeclamptic women were found to have significantly higher levels of creatinine (97.13 umol/L), urea (6.87 mmol/L) and uric acid (478.98 mg/dL) compared to normotensives with creatinine, urea and uric acid of 60.84 umol/L, 3.61 mmol/L and 275.66 mg/dL respectively (*p* < 0.001) (Table 3).

**Table 3:** Biochemical characteristics of study participants.

Variable	Normotensive (n = 75)	Preeclampsia (n = 75)	*p* value
Mg (mmol/L)	0.82 ± 0.12	0.75 ± 0.13	**< 0.0001**
Na (mmol/L)	140.76 ± 2.52	137.22 ± 2.45	**< 0.0001**
Cl (mmol/L)	102.86 ± 1.85	101.56 ± 2.02	**< 0.0001**
Creatinine (umol/L)	60.84 (56.77–64.33)	97.13 (89.78–102.87)	**< 0.0001**
Urea (mmol/L)	3.61 (3.09–3.97)	6.87 (5.89–7.91)	**< 0.0001**
K (mmol/L)	4.13 (3.87–4.66)	3.76 (3.65–3.92)	**< 0.0001**
Uric acid (mg/dL)	275.66 (269.02–280.91)	478.98 (471.79–483.46)	**< 0.0001**

Mg: Magnesium, Na: Sodium, Cl: Chloride, K: Potassium.

*p* < 0.05 and bolded means statistically significant.

**Figure 2:**
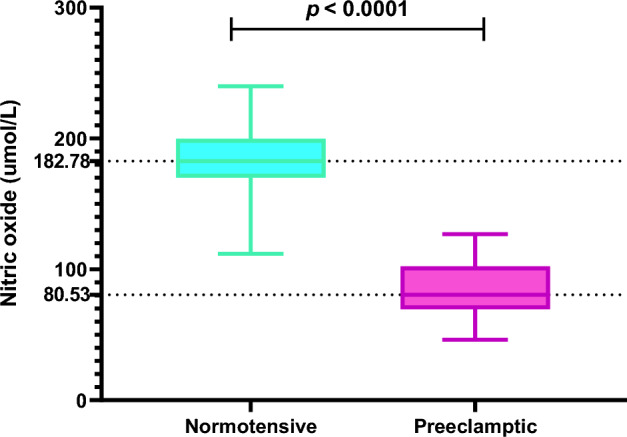
Plasma nitric oxide concentration among normotensive and preeclamptic women.

Preeclamptic patients had significantly lower nitric oxide concentration compared to the normotensives (*p* < 0.0001) (Fig. 2).

### VNTR allele frequencies, T786C allele frequencies and genotype distributions in normotensive and preeclamptic pregnant women

**Figure 3:**
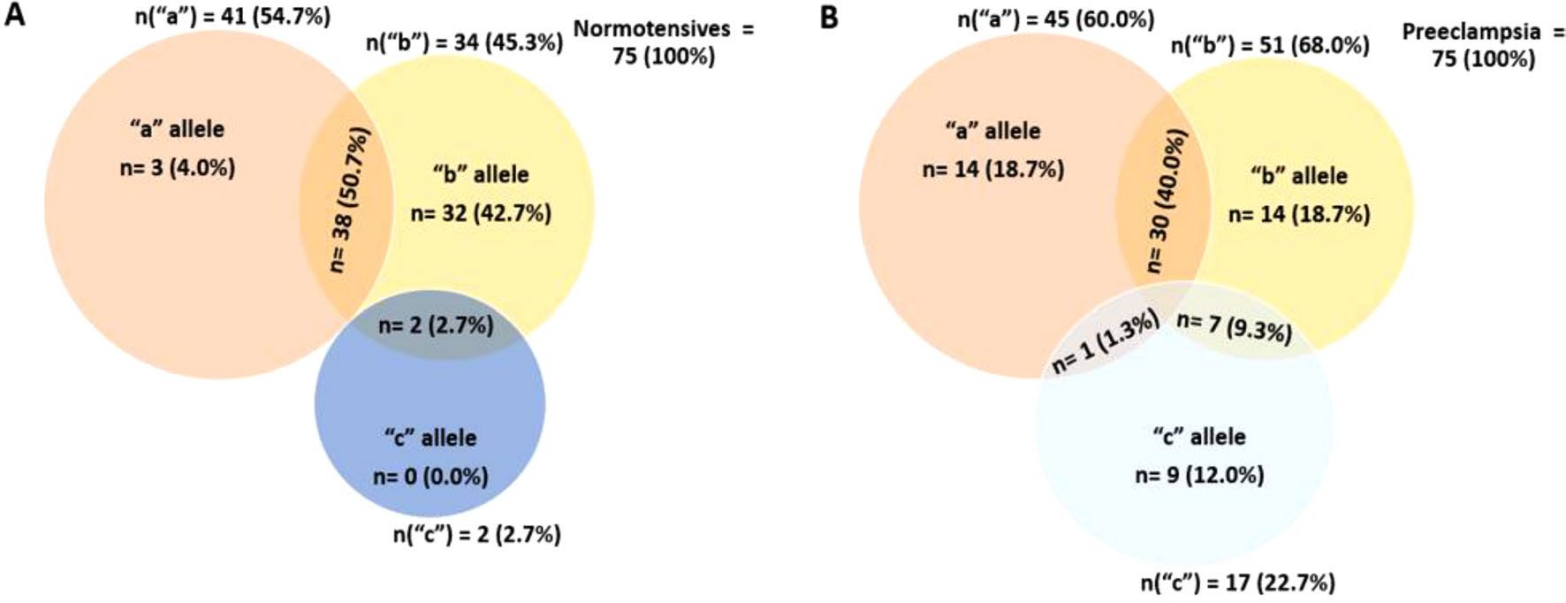
VNTR allele frequency distribution in normotensive (**A**) and preeclamptic (**B**) pregnant women.

The VNTR allele distribution between the preeclamptic and normotensives women showed that, the normotensives had complete absence of “4c” allele (0.0), whilst the preeclamptic women had a significant number of “4c” allele (12.0%). The “4b” allele (42.7%) was predominantly found in the normotensives, whereas the preeclamptic women had a proportional number of “4a” allele (18.7%) and “4b” allele (18.7%) (Fig. 3).

**Figure 4:**
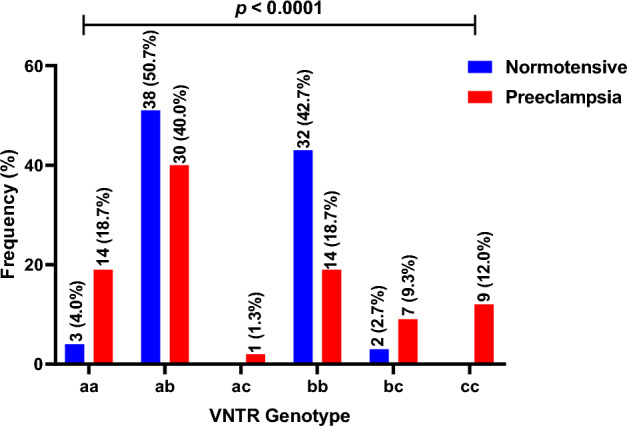
VNTR intron 4 genotype distribution in normotensive and preeclamptic pregnant women.

The VNTR genotype distribution between the preeclamptic and normotensives women showed that, “4c4c” (12.0%) and “4a4c” (1.3%) genotypes were found predominantly in preeclamptic women. Moreover, “4a4a” and “4b4c” genotypes, majority were found among preeclamptic women (“4a4a”: 14.7%, “4b4c”: 9.3%) compared to the normotensives (“4a4a”: 4.0%, “4b4c”: 2.7%). However, for the “4a4b” and “4b4b” genotypes, lower prevalence was observed in preeclamptic women (“4a4b”: 40.0%, “4b4b”: 18.7%) compared to normotensives (“4a4b”: 50.7%, “4b4b”: 42.7%). A significant difference in VNTR intron 4 distribution was therefore observed between the preeclamptic and normotensives women (*p* < 0.0001) (Fig. 4).

**Figure 5:**
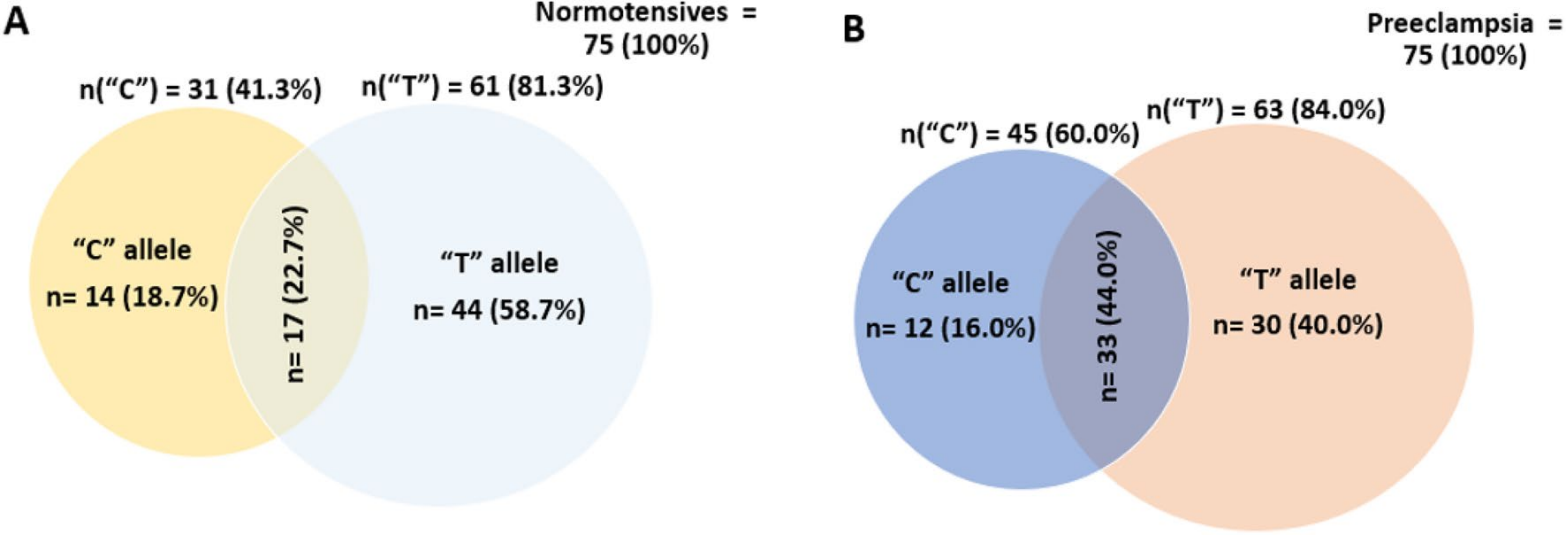
T786C allele frequency distribution in normotensive (**A**) and preeclamptic (**B**) pregnant women.

The distribution of T786C alleles was almost proportional among preeclamptic and normotensives women. Thus, both preeclamptic and normotensive women had predominantly “T” allele (58.7% vs 40.0%), followed by “C” allele (18.7% vs 16.0%) (Fig. 5).

**Figure 6:**
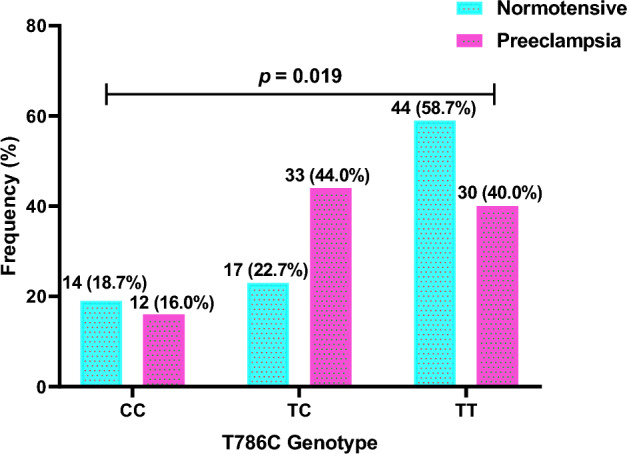
T786C genotype distribution in normotensive and preeclamptic pregnant women.

The findings from T786C genotype distribution revealed that, a higher frequency of “TC” genotype was found in preeclamptic women (44.0%) compared to normotensives (22.7%). A lower frequency was however observed for “CC” genotype in preeclamptic women (16.0%) compared to normotensives (18.7%). Similarly, “TT” genotype frequency was lower in preeclamptic women (40.0%) compared to normotensives (58.7%). Thus, the distribution of T786C allele distribution was significantly different between normotensives and preeclamptic pregnant women (*p* = 0.019) (Fig. 6).

### Association between VNTR variants, T786C variants and differences in nitric oxide concentration in preeclampsia

**Figure 7:**
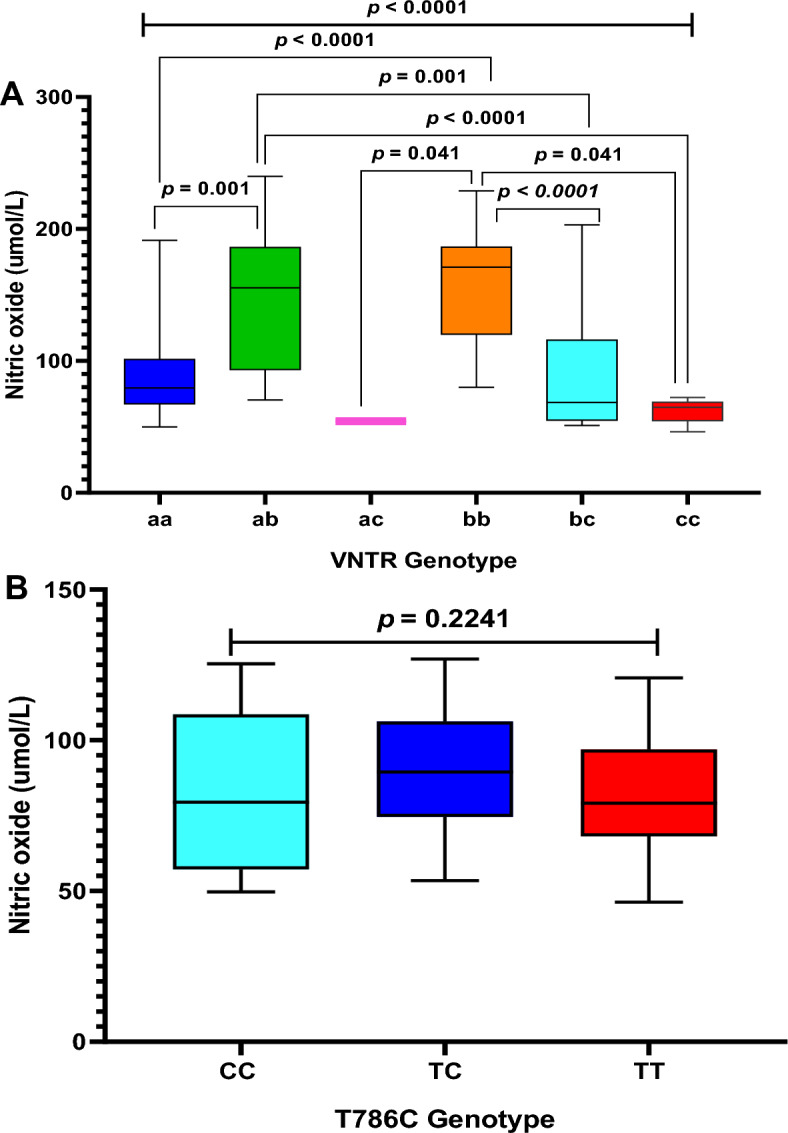
Association between VNTR variants (**A**) and T786C variants (**B**) and nitric oxide in preeclampsia.

Nitric oxide concentrations were found to be significantly different between preeclamptic women with different VNTR variants (*p* < 0.0001). In a post-hoc test, nitric oxide concentrations were significantly different between women with allele of “4a4a” and that of “4a4b” (*p* < 0.001), and “4b4b” (*p* < 0.0001). Again, a significant difference in levels of nitric oxide concentration was observed between women with “4a4b” allele and that of “4b4c” (*p* < 0.001), and “4c4c” (*p* < 0.0001). Similarly, levels of nitric oxide concentrations were significantly different between women with “4b4b” genotype and that of “4a4c” (*p* < 0.041), “4b4c” (*p* < 0.0001) and “4c4c” (*p* < 0.041) (Fig. 7A).

On the contrary, the comparison of T786C variant distribution and nitric oxide concentrations level showed that, there was no significant difference between levels of nitric oxide concentrations and various T786C variant in preeclamptic women (*p* = 0.2241) (Fig. 7B).

### Association between VNTR and T786C variants and risk of preeclampsia

Our study reveals that, compared to preeclamptic women with “4a4a” VNTR genotype, those with “4a4b” (cOR: 0.17, 95% CI: 0.04–0.64) variant had 83% decreased chance of experiencing preeclampsia. Moreover, compared to preeclamptic women with “4a4a” VNTR genotype, those with “4b4b” (cOR: 0.09, 95% CI 0.02–0.38) variant had 91% decreased likelihood of experiencing preeclampsia (Table 4). Thus, possessing “4b” as an allele in the VNTR variant genotype decreases the risk of preeclampsia.

**Table 4:** Association between VNTR and T786C variants and risk of preeclampsia.

Variable	Preeclampsia (n = 75)	cOR (95% CI)	*p* value
VNTR genotype			
4a4a	14 (18.7)	1.00	–
4a4b	30 (40.0)	0.17 (0.04–0.64)	**0.0090**
4a4c	1 (1.3)	346,173,180.6 (0.00-inf)	> 0.9999
4b4b	14 (18.7)	0.09 (0.02–0.38)	**0.0010**
4b4c	7 (9.3)	0.75 (0.01–5.58)	0.7790
4c4c	9 (12.0)	346,173,180.6 (0.00-inf)	0.9990
T786C genotype			
CC	12 (16.0)	1.00	-
TC	33 (44.0)	2.27 (0.86–5.96)	0.0980
TT	30 (40.0)	0.80 (0.32–1.96)	0.6180

*p* < 0.05 and bolded means statistically significant.

There was however no significant association between T786C variants and the risk of preeclampsia for either “TC” genotype (cOR: 0.2.27, 95% CI: 0.86–5.96; *p* = 0.0980) or “TT” genotype (cOR: 0.0.80, 95% CI: 0.32–1.96; *p* = 0.6180) compared to the “CC” genotypes (Table 4)**.**

## Discussion

Preeclampsia remains a leading cause of maternal morbidities and mortalities worldwide. Our previous study has reported that decreased bioavailability of nitric oxide plays a vital role in preeclampsia^10^. In this current study, we assessed the association between eNOS gene polymorphism and nitric oxide levels among Ghanaian preeclamptic women. This study found that, nitric oxide (NO) is reduced in preeclamptic women. Moreover, NO in preeclampsia is altered by VNTR intron 4 variants but not T786C variants; and also possessing VNTR intron 4 “4b” allele decreases the risk of PE whilst “4c” allele increases the risk of PE. The study further revealed that compared to 4a4a, VNTR genotypes 4b4b and 4a4b reduced preeclampsia risk by 91% and 83% respectively. Generally, the ‘4b’ allele was associated with increased nitric oxide concentrations in comparison with the ‘4a’ and ‘4c’ alleles thus reducing ‘4b’ carriers’ risk of preeclampsia.

Similar to other studies^10,11,15^ we observed that preeclamptic women have significantly lower nitric oxide concentration compared to the normotensive pregnant women. NO is a regulator of endothelial function controlling the tone of the vasculature and blood flow through the stimulation of guanylate cyclase in the smooth muscle^17^. Reduction in its bioavailability triggers vascular pathologies with resulting complications such as preeclampsia and atherosclerosis^18^. Moreover, variants of the eNOS gene could alter or slow down transcriptional and translational processes in preeclampsia leading to reduction in NO bioavailability.

Evaluation of the VNTR intron 4 allele distributions in the current study demonstrated the absence of homozygous “4c’’ allele in the genotypes of normotensive pregnant women whereas significantly present in preeclampsia. The “4b” allele was predominantly found in the normotensives. Our findings on the VNTR allele distribution in the study are consistent with studies by Thomas et al.^19^ and Tanus-Santos et al*.*^20^ conducted among Africans, Caucasians and African-Americans. They reported that 4c allele is rare as compared to the commonly encountered 4a and 4b, though evidence has shown that it is common in people with African descent. This buttresses our finding of 4c allele in the Ghanaian population where all the respondents are of the African descent. Sigusch et al*.*^21^ found the 4c allele in people with coronary artery disease and hypertension suggesting its relevance to cardiovascular diseases. The presence of 4c allele among preeclamptic women in this study could be attributed to a potential role of this intronic allele in decreasing transcript levels by modulating transcription rate and stability as well as reducing translation efficiency leading to decrease in NO. A significant difference in VNTR intron 4 distribution was also observed between the normotensives and preeclamptic women with 4c4c, 4a4c, 4b4c and 4a4a being predominant in preeclamptic women as compared to the normotensives. Similar results were reported in other studies^11,22,23^. Groten et al*.*^23^ similarly found that carriers of eNOSI_4_ VNTR4a had 1.7-increased risk of developing preeclampsia. Shaheen et al*.*^24^ conversely did not find a significant difference in VNTR intron 4 genotype distribution between preeclamptic and normotensive pregnant women^24^. Geographic and ethnic differences in their study population could account for the dissimilar findings.

The distribution of T786C alleles was proportional between preeclamptic and normotensives women. This is in conformity to previous work by Alpoim et al*.*^22^ who also did not find any significant difference in T786C alleles between normotensives and preeclamptic women. T786C genotype distribution in this current study showed a higher frequency of “TC” genotype in preeclamptic women compared to normotensives. However, the study found T786C genotype distribution to be significantly different between normotensives and preeclamptic pregnant women as reported by Shaheen et al*.*^24^. In contrast, our finding does not conform to findings from studies conducted by Sandrim et al*.*^11^ and Chen et al*.*^15^ in Brazil and China respectively. Their studies found no significant difference in T786C allele distribution between normotensive pregnant women and preeclamptic women. This discrepancy may be explained by the ethnic differences among study participants.

VNTR intron 4 variants significantly modified nitric oxide levels in the preeclamptic women, however, there was no significant difference between levels of nitric oxide and various T786C variants in preeclamptic women. Thus, nitric oxide concentrations were significantly different among preeclamptic women with different VNTR variants. NO levels were higher in preeclamptic women with the “4b4b” genotype as compared to those with “4a4a” genotype in our study. Alpoim et al*.*^22^ reported a similar finding in Brazil where subjects with homozygous “4a” allele had reduced NO concentrations. Generally, preeclamptic subjects with “4a4a”, “4a4c” and “4c4c” had reduced NO concentrations as compared to the other VNTR intron 4 genotypes; “4b4b”, “4b4c” and “4a4b”. Thus the “4a” and “4c” alleles may alter the splicing of messenger RNA transcripts of the eNOS gene leading to significant modifications of the gene function consequently causing reduction of NO metabolites. Chen et al*.*^11^ however found preeclamptic women with “4a4a” genotype to have higher NO concentrations as compared to their “4b4b” counterparts. Ethnic origin of the studied populations may explain the divergence observed as suggested in previous study^25^. Our study population were all Ghanaians and for that matter Africans. The similarity of our work with the study in Brazil by Alpoim et al*.*^22^ may likely be as a result of the rather heterogeneous population of the country where a lot of native Brazilians have African ancestry. In the study conducted in mainland China^11^, all subjects were from the Han population who are all Asians and may therefore account for the dissimilarities observed between our study and theirs.

Furthermore, we observed that possessing “4b” as an allele in a VNTR intron 4 genotype decreases the risk of preeclampsia while the “4c” allele increases the risk of preeclampsia. Our study demonstrated that compared to preeclamptic women with “4a4a” VNTR genotype, those with “4b4b” variant had 91% decreased likelihood of experiencing preeclampsia. This is similar to study by Jakovljevic et al.^26^ where they found an eightfold increased risk of PE for homozygous 4a4a patients when compared to 4b4b subjects. Groten et al.^23^, in a study involving Caucasians and African women, particularly from Ghana, also found that ‘4b’ carriers had decreased risk of preeclampsia in the total cohort which is in conformity with the present study. This suggest that the finding that possessing ‘4b’ allele with its associated reduced risk of preeclampsia could hold true for different populations. Although, a few case–control studies conducted on VNTR intron 4 polymorphisms and their association with PE have reported no association in other populations. Ozturk et al*.*^27^ found that there was no association between variants and risk of PE in a Turkish population explaining that their location as intronic alleles may not pose any risk to the development of preeclampsia. On the contrary, the present study suggests that “4a” and “4c” alleles may contribute significantly to endothelial dysfunction by reducing NO levels leading to preeclampsia. It is probable the wild type allele (4b) with five 27 bp repeats does not significantly reduce the eNOS gene expression as compared to the 4c and 4a alleles with six and four 27 bp repeats respectively.

This study which evaluated the association between variants of the eNOS gene and nitric oxide production in women with preeclampsia has established association between VNTR variants and nitric oxide levels but not T786C variants. The study is limited by the unavailability of data on eNOS enzyme estimation as well as postpartum nitric oxide concentration of the study participants. However, our study provides very significant findings on the eNOS gene variants and nitric oxide production in preeclampsia.

## Conclusion

Nitric oxide (NO) is reduced in preeclamptic women. NO levels in preeclampsia are altered by VNTR intron 4 variants but not T786C variants. Possessing VNTR intron 4 “4b” allele decreases the risk of PE while the “4c” allele increases the risk of preeclampsia. There is the need for eNOS variant screening and nitric oxide estimation among pregnant women for early prediction of women at risk of preeclampsia. This may also be useful in identifying subjects who will benefit from NO-donors during pregnancy. Prospective longitudinal study could evaluate NO levels in preeclamptic women ante- and postpartum.

## Data Availability

All data generated or analyzed during this study are within this manuscript.
